# Association between the inflammatory burden index and rheumatoid arthritis and its all-cause mortality: data from NHANES 1999–2018

**DOI:** 10.3389/fmed.2024.1421497

**Published:** 2024-08-21

**Authors:** Jiali Zhai, Bo Yuan, Tiebing Liu, Linfei Mo, Yajie Xie, Yi Zhao, Shuai Cao, Liesu Meng

**Affiliations:** ^1^Department of Biochemistry and Molecular Biology, Institute of Molecular and Translational Medicine (IMTM), Xi'an Jiaotong University Health Science Center, Xi'an, China; ^2^Department of Orthopedics, Civil Aviation General Hospital, Beijing, China; ^3^Civil Aviation Public Health Emergency Management Office, Civil Aviation Medicine Center, Civil Aviation Administration of China, Civil Aviation General Hospital, Beijing, China; ^4^Department of Rheumatology and Immunology, First Affiliated Hospital of Xi’an Jiaotong University, Xi'an, China; ^5^Department of Rheumatology and Immunology, and Clinical Institute of Inflammation and Immunology, Frontiers Science Center for Disease-Related Molecular Network, West China Hospital, Sichuan University, Chengdu, China; ^6^Key Laboratory of Environment and Genes Related to Diseases, Xi'an Jiaotong University, Ministry of Education, Xi'an, China

**Keywords:** rheumatoid arthritis, inflammatory burden index, all-cause mortality, national health and nutrition examination survey, non-linear

## Abstract

**Background and aims:**

Rheumatoid arthritis (RA) is a prevalent chronic autoimmune disease characterized by chronic inflammation. The Inflammatory Burden Index (IBI) is a newly proposed comprehensive inflammation index used to assess systemic inflammation. The relationship between IBI and RA, as well as its all-cause mortality, remains unclear. The objective of this study was to examine the correlation between IBI and RA and to analyze the association between IBI and all-cause mortality in RA.

**Methods:**

The study comprehensively analyzes adult data from the National Health and Nutrition Examination Survey (NHANES) spanning 1999 to 2018. The participants’ IBI was calculated using the formula IBI = CRP * neutrophils/lymphocytes. Three models were constructed to investigate the relationship between IBI and the prevalence of RA. Nonlinear relationships were determined using restricted cubic spline curves. Stratified analyses and interaction tests were used to explore the relationship between RA and IBI in different subgroups. The same data analyses were applied to investigate the association between IBI and RA all-cause mortality.

**Results:**

The data analyses revealed a stable positive and nonlinear correlation between IBI and the risk of RA, as well as a positive, nonlinear, J-shaped association between IBI and RA all-cause mortality. The correlation and association were consistent across most subgroups, and multiple covariates had no effect on the results. No significant effect of multiple covariates on the association was found through interaction tests.

**Conclusion:**

Our study has demonstrated a positive correlation between the prevalence of RA and all-cause mortality with the IBI index. This suggests that lower levels of inflammation in the body are associated with a reduced risk of RA prevalence and all-cause mortality. Further prospective studies are required to explore the mechanisms involved.

## 1 Introduction

Rheumatoid arthritis(RA), the most prevalent systemic autoimmune disease, is typically characterized by progressive symmetrical inflammation of the affected joints (1). Joint swelling in RA is mainly due to synovial inflammation resulting from activation of the immune system and consequent infiltration of leukocytes into the synovial compartment (2). The chronic inflammatory state of the patient leads to extra-articular manifestations, including rheumatoid nodules and vasculitis, in addition to cardiovascular, pulmonary, neurological, gastrointestinal, renal, and hematological disorders (3). This ultimately leads to an elevated incidence of deformity disability and may contribute to early death (4). Specifically, during the course of RA, there is an increase in the number of innate and acquired immune cells and a persistent chronic inflammatory profile (5).

Variations in a panel of inflammatory markers, such as neutrophils, lymphocytes, platelets, and C-reactive protein (CRP), are routinely used to assess the state of systemic inflammation. The Inflammatory Burden Index (IBI) is a newly proposed comprehensive index to evaluate systemic inflammation. It is derived from the CRP*neutrophils/lymphocytes formula (6). The IBI can be applied to assess the inflammatory load of a variety of diseases and to predict the prognosis of patients (7, 8).

Research has shown that certain inflammatory markers, including CRP and neutrophil-to-lymphocyte ratio, are positively associated with the risk of all-cause mortality in the general population (9, 10). However, it is unclear whether there is a relationship between IBI as a comprehensive inflammatory marker and RA, as well as its all-cause mortality. To address this issue, we conducted analyses using the National Health and Nutrition Examination Survey (NHANES) database, a comprehensive and well-respected database known for its robust methodology and wide range of health data. It has been utilized in numerous international studies to explore health trends and associations of global relevance. The present study analyzed data from NHANES conducted between 1999 and 2018 to investigate the correlation between IBI and RA, and examine the association between IBI and all-cause mortality in RA.

## 2 Method

### 2.1 Study population

This study utilized data from NHANES, a survey project conducted by the National Center for Health Statistics, to evaluate the health and nutritional status of the US population between 1999 and 2018. NHANES is a nationally representative cross-sectional study that collects statistical information biennially. Data are refined and collected in two primary ways. The study collected detailed information on demographics, dietary factors, and health status of the population through household interviews. Additionally, a standard medical physical examination was conducted at a dedicated Mobile Examination Centre while biological samples were collected for assays. NHANES received approval from the Institutional Research Ethics Review Board of the National Center for Health Statistics, and all participants provided written informed consent.

**Figure 1:**
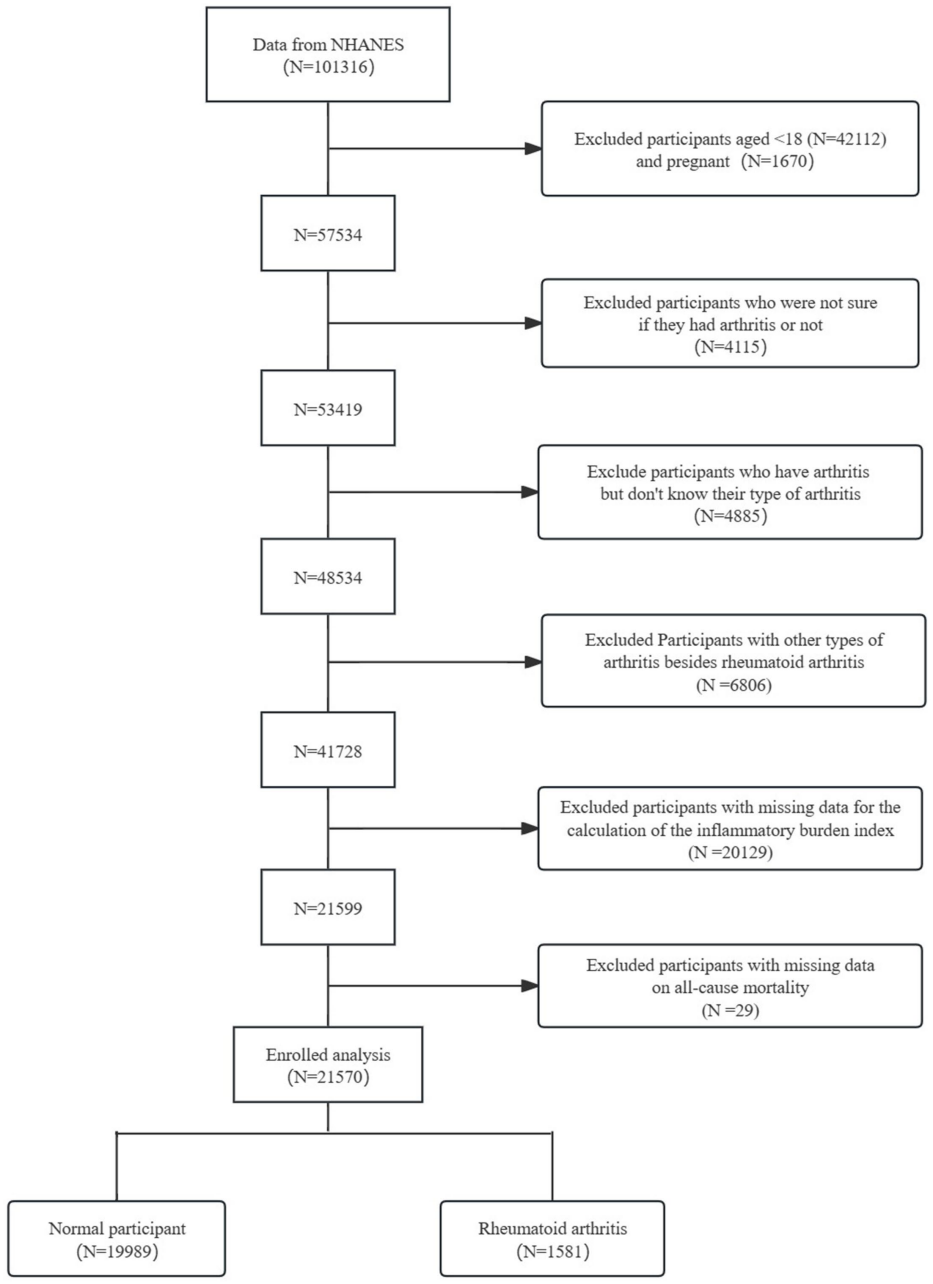
Flowchart of the participant selection from NHANES 1999–2018.

The study included 101,316 participants from the NHANES. Participants were screened based on their questionnaire and laboratory test results. The screening process aimed to (1): Participants under 18 years of age (*N* = 42,112) and pregnant women (*N* = 1,670) were excluded (2). Participants uncertain whether they had arthritis (*N* = 4,115) were excluded (3). Participants with arthritis but unknown type were excluded (*N* = 4,885) (4). Participants with arthritis types other than RA were excluded (*N* = 6,806) (5). Participants with missing data for calculating the IBI were excluded (*N* = 20,129) (6). Participants with missing data on all-cause mortality were excluded (*N* = 29). The final study population comprised 21,570 participants, including 1,581 with RA and 19,989 normal controls (Figure 1).

### 2.2 Assessment of RA

RA was assessed through a self-report questionnaire. Participants make a response to a question on the questionnaire: whether a doctor or other health professional has told them they have arthritis. If participants opted for “yes, “they answered the next follow-up question: what type of arthritis do they have? Participants were categorized into either the RA group or the non-RA group by their answers to this question. Participants were enrolled in the survey if they answered “yes” to the first question and “RA” to the second question.

### 2.3 IBI

IBI was derived from three indicators: neutrophils, lymphocytes, and CRP, and was calculated from the participants’ laboratory measurements using CRP multiplied by neutrophils divided by lymphocytes. The formula is IBI = CRP * neutrophils/lymphocytes (6). Higher calculated IBI scores denoted a higher inflammatory burden in participants; inversely, lower IBI scores signaled a lower inflammatory burden in participants.

### 2.4 Outcome

This study examines the relationship between IBI and all-cause mortality in RA by exploring the association between IBI and RA. All-cause mortality refers to death from any cause. Mortality status information was obtained by probabilistic matching using individual identifiers to link NHANES data to the National Death Index. Follow-up time was calculated from the NHANES interview to the date of death.

### 2.5 Covariates

Covariates were used to account for potential confounding factors that could influence the analysis results. A household interview questionnaire was used to collect socio-demographic data from participants, including age, gender, race, education level, marital status, household poverty-to-income ratio (PIR), smoking status, alcohol consumption, and disease status (cardiovascular disease (CVD), diabetes mellitus (DM), hypertension, RA, and stroke). Additionally, physical activity and recreational activity were assessed. The study categorized race as Mexican American, non-Hispanic Black, non-Hispanic White, and other. Educational attainment was categorized as less than high school, high school graduate/equivalent and other. Marital status was categorized as married/living with a partner, and other. Household PIR, an indicator of poverty, was derived by dividing household income by a poverty indicator and was categorized as <1.3, 1.3 ≤ PIR<3.5, and PIR ≥ 3.5. Smoking status was categorized as never, former, or current, while alcohol consumption status was categorized as never, mild, moderate, heavy, or former. The laboratory measured serum glycated hemoglobin (HbA1c), alanine aminotransferase (ALT), aspartate aminotransferase (AST), total cholesterol (TC), high-density lipoproteins (HDL), low-density lipoproteins (LDL), and blood urea nitrogen (BUN) using standard methods. At the Mobile Examination Centre, height and weight were measured to calculate body mass index (BMI). BMI was calculated by dividing weight in kilograms by the square of height in meters. The study participants were categorized according to BMI as <18.5, 18.5 ≤ BMI < 25, and BMI ≥ 25. Strict quality control was implemented during blood collection and analysis.

### 2.6 Statistical analysis

The NHANES database utilizes advanced survey designs to reduce data bias resulting from statistical procedures. Therefore, in this study, the sample data was weighted using the sample weighting method to produce nationally representative statistics. The IBI scores were divided into four quartiles. Weighted means and 95% confidence intervals were used to express continuous variables and analyzed with weighted linear regression. Categorical variables were expressed as weighted percentages with 95% confidence intervals and analyzed using weighted chi-square tests.

The relationship between IBI and RA prevalence was analyzed using regression analysis. Three multivariate models were constructed to explore this relationship, each adjusting for distinct confounders. Model 1 did not adjust for any confounders, while Model 2 adjusted for age, gender, race, education, marital status, household PIR, BMI, smoking status, drinking status, stroke, CVD, DM, and hypertension. Model 3 was adjusted for age, sex, race, education, marital status, household PIR, BMI, smoking status, drinking status, stroke, CVD, DM, hypertension, HbA1c, ALT, AST, BUN, TC, and HDL. Additionally, we evaluated the dose–response relationship between IBI and RA using restricted cubic spline (RCS) curves. Furthermore, we conducted stratified analyses and interaction tests as sensitivity analyses to further investigate the relationship between IBI and RA in different subgroups, including age, sex, race, education, marital status, household PIR, BMI, smoking status, drinking status, stroke, CVD, DM, hypertension, HbA1c, ALT, AST, BUN, TC, and HDL.

The study categorized RA patients into mortality and non-mortality groups based on outcome. The association between IBI and all-cause mortality in the RA mortality group was analyzed. Three models were constructed to explore the association between IBI and all-cause mortality, adjusting for different confounders. The models were adjusted for the same covariates as above. The dose–response relationship between IBI and RA all-cause mortality was investigated using RCS curves. Stratified analyses and interaction tests were conducted to explore whether confounding factors affected the stability of the results.

The correlation between IBI and the risk of developing RA and its all-cause mortality is to be explored through the multiple statistical methods described above. All statistical analyses were performed using R software. The statistical tests were two-sided, and a two-tailed *p*-value of less than 0.05 indicated a statistically significant result.

## 3 Results

### 3.1 Baseline characteristics according to quartiles of IBI

Table 1 outlines the characteristics of the study population, which consisted of 21,570 adult participants from the US. The demographic and clinical features of the participants were statistically represented by quartiles of IBI scores. Table 1 presents the results, which indicate statistically significant differences in demographics, including age, gender, race, education level, marital status, smoking status, alcohol consumption, household PIR, BMI, CVD, DM, hypertension, RA, stroke, and recreational activities, as well as in blood tests such as HbA1c, ALT, AST, LDL, HDL, TC, and BUN (*p* < 0.05). Participants who were female, non-Mexican white, highly educated, married/partnered, non-smokers, mild alcohol drinkers, with high household PIR and high BMI, and without CVD, diabetes, hypertension, or RA had higher IBI levels. As IBI scores increased, HbA1c, ALT, AST, LDL, BUN, and TC levels increased, while HDL levels decreased. AS IBI scores increased, the prevalence of CVD, DM, hypertension, and RA increased.

**Table 1:** The demographic and clinical characteristics of the patients by quartiles of baseline inflammatory burden index (IBI).

Characteristics	IBI				*p*-value
	Q1	Q2	Q3	Q4	
*N*	5,392	5,393	5,392	5,393	
Age (years)	39.919 (39.424,40.415)	43.689 (43.134,44.244)	45.476 (44.935,46.017)	46.510 (45.949,47.072)	<0.001
Gender (%)					<0.001
Female	44.219 (42.574,45.876)	43.355 (41.839,44.884)	49.638 (47.944,51.332)	59.271 (57.622,60.899)	
Male	55.781 (54.124,57.426)	56.645 (55.116,58.161)	50.362 (48.668,52.056)	40.729 (39.101,42.378)	
Race (%)					<0.001
Mexican American	7.618 (6.597,8.781)	9.083 (7.733,10.641)	8.940 (7.482,10.649)	9.547 (7.715,11.759)	
Non-Hispanic Black	11.726 (10.315,13.301)	10.032 (8.696,11.547)	10.352 (8.972,11.917)	12.176 (10.659,13.874)	
Non-Hispanic White	67.800 (65.216,70.279)	69.543 (66.541,72.386)	69.645 (66.738,72.403)	69.024 (65.722,72.142)	
Other	12.857 (11.269,14.631)	11.343 (9.395,13.635)	11.063 (9.319,13.087)	9.254 (7.709,11.071)	
Education (%)					<0.001
Less Than High School	5.332 (4.719,6.020)	6.484 (5.784,7.262)	6.566 (5.937,7.256)	6.732 (5.868,7.713)	
High School Grad/GED or Equivalent	32.435 (30.513,34.418)	35.715 (33.853,37.622)	39.456 (37.523,41.423)	41.210 (39.498,42.944)	
Other	62.233 (60.190,64.233)	57.801 (55.734,59.841)	53.978 (51.912,56.032)	52.058 (50.245,53.865)	
Marital status (%)					<0.001
Married/Living with partner	63.455 (61.529,65.339)	65.475 (63.620,67.283)	66.702 (65.090,68.276)	61.495 (59.631,63.326)	
Other	36.545 (34.661,38.471)	34.525 (32.717,36.380)	33.298 (31.724,34.910)	38.505 (36.674,40.369)	
Smoking status (%)					<0.001
Never	57.156 (55.193,59.097)	52.968 (51.132,54.797)	51.366 (49.431,53.297)	49.087 (46.864,51.313)	
Former	21.238 (19.841,22.705)	22.694 (21.213,24.246)	22.940 (21.418,24.536)	23.700 (22.052,25.431)	
Now	21.606 (20.165,23.122)	24.338 (22.804,25.941)	25.694 (23.946,27.523)	27.213 (25.750,28.728)	
HbA1c (%)	5.280 (5.258,5.302)	5.407 (5.380,5.434)	5.527 (5.498,5.556)	5.676 (5.640,5.711)	<0.001
Drinking alcohol status (%)	10.837 (9.011,12.980)	10.293 (8.794,12.015)	11.308 (9.767,13.057)	12.803 (11.334,14.431)	<0.001
Never	36.830 (34.951,38.750)	36.032 (33.854,38.269)	33.142 (30.937,35.424)	29.507 (27.531,31.564)	
Mild	17.323 (15.906,18.838)	16.170 (14.819,17.619)	16.382 (14.919,17.960)	15.728 (14.423,17.128)	
Moderate	23.696 (22.040,25.436)	23.400 (21.518,25.394)	22.739 (21.133,24.430)	22.437 (20.908,24.044)	
Heavy	11.314 (10.268,12.451)	14.105 (12.728,15.605)	16.428 (14.977,17.989)	19.524 (18.033,21.107)	
Former					
Family PIR	18.232 (16.722,19.846)	19.034 (17.557,20.603)	20.375 (18.803,22.043)	24.355 (22.617,26.181)	<0.001
<1.3	34.117 (32.288,35.995)	35.348 (33.353,37.396)	36.854 (35.064,38.681)	37.085 (35.105,39.109)	
≥1.3, <3.5	47.651 (45.111,50.202)	45.618 (43.008,48.253)	42.771 (40.601,44.969)	38.560 (36.181,40.995)	
≥3.5					
BMI (kg/m^2^)					<0.001
<18.5	4.103 (3.554,4.733)	1.580 (1.187,2.099)	1.019 (0.703,1.475)	0.748 (0.524,1.066)	
≥18.5, <25	53.542 (51.730,55.345)	34.833 (33.239,36.462)	24.127 (22.632,25.689)	17.701 (16.339,19.151)	
≥25	42.355 (40.491,44.241)	63.588 (61.894,65.249)	74.854 (73.254,76.389)	81.551 (80.001,83.006)	
DM (%)					<0.001
No	95.836 (95.190,96.398)	93.941 (93.284,94.538)	90.993 (90.111,91.803)	86.742 (85.645,87.767)	
Yes	4.164 (3.602,4.810)	6.059 (5.462,6.716)	9.007 (8.197,9.889)	13.258 (12.233,14.355)	
CVD (%)					<0.001
No	96.275 (95.639,96.822)	95.264 (94.572,95.872)	92.825 (91.869,93.676)	89.719 (88.773,90.594)	
Yes	3.725 (3.178,4.361)	4.736 (4.128,5.428)	7.175 (6.324,8.131)	10.281 (9.406,11.227)	
ALT(U/L)	23.977 (23.535,24.419)	26.672 (26.169,27.174)	28.101 (27.347,28.856)	27.337 (25.990,28.685)	<0.001
AST(U/L)	24.676 (24.376,24.977)	25.425 (25.056,25.793)	26.187 (25.566,26.807)	25.539 (24.904,26.174)	<0.001
Hypertension (%)					<0.001
No	80.152 (78.716,81.514)	71.959 (70.504,73.369)	65.467 (63.894,67.006)	58.498 (56.615,60.356)	
Yes	19.848 (18.486,21.284)	28.041 (26.631,29.496)	34.533 (32.994,36.106)	41.502 (39.644,43.385)	
RA (%)					<0.001
No	97.203 (96.676,97.649)	95.683 (94.969,96.300)	94.259 (93.521,94.917)	90.579 (89.662,91.422)	
Yes	2.797 (2.351,3.324)	4.317 (3.700,5.031)	5.741 (5.083,6.479)	9.421 (8.578,10.338)	
Stroke					<0.001
No	99.083 (98.777,99.313)	98.491 (98.120,98.790)	97.918 (97.469,98.289)	96.854 (96.230,97.378)	
Yes	0.917 (0.687,1.223)	1.509 (1.210,1.880)	2.082 (1.711,2.531)	3.146 (2.622,3.770)	
LDL (mmol/L)	112.193 (110.499,113.887)	119.869 (118.077,121.661)	121.332 (119.834,122.829)	119.272 (117.041,121.502)	<0.001
HDL (mmol/L)	56.253 (55.622,56.884)	52.418 (51.875,52.961)	50.288 (49.700,50.875)	49.456 (48.906,50.006)	<0.001
TC (mmol/L)	190.194 (188.849,191.539)	199.974 (198.536,201.411)	203.678 (202.080,205.276)	200.264 (198.562,201.966)	<0.001
BUN (mmol/L)	12.665 (12.515,12.816)	12.915 (12.748,13.081)	13.038 (12.846,13.230)	13.023 (12.834,13.212)	0.001
Physical activity (MET)	3217.652 (2934.953,3500.351)	3493.176 (3203.674,3782.679)	3274.999 (2997.509,3552.489)	3123.722 (2767.587,3479.858)	0.154
Recreational activity(MET)	1759.616 (1649.554,1869.679)	1664.508 (1520.994,1808.021)	1543.790 (1423.237,1664.342)	1246.351 (1138.599,1354.103)	<0.001

HbA1c, glycated hemoglobin; PIR, poverty-to-income ratio; BMI, body mass index; DM, diabetes; CVD, cerebrovascular disease; ALT, alanine aminotransferase; AST, aspartate aminotransferase; RA, rheumatoid arthritis; LDL, low-density lipoprotein; HDL, high-density lipoprotein; TC, total cholesterol; BUN, blood urea nitrogen.

### 3.2 Relationship between IBI and the risk of RA

Table 2 shows the results of regression analyses between IBI and RA prevalence risk obtained from different model tests. Notably, RA prevalence was positively and significantly correlated with the IBI score in all three models (*p* < 0.05). The IBI was converted into quartiles, and the highest quartile was associated with an increased risk of RA compared to the lowest quartile. In Model 1, participants in the highest quartile had a significantly higher odds ratio (OR) for RA prevalence compared with participants in the lowest IBI quartile (OR for Q2, 1.554 [95% CI: 1.295, 1.864]; for Q3, 2.266 [95% CI: 1.909, 2.691]; for Q4, 3.471 [95% CI: 2.948, 4.087]). In Model 2, RA prevalence was 72.3% higher in the highest quartile than in the lowest quartile (Q4, 1.723 [95% CI: 1.422, 2.087]), which was a statistically significant difference (*p* < 0.05). In Model 3, adjusted for all covariates, the prevalence of RA was 72.1% higher in the highest quartile group than in the lowest quartile group (Q4, 1.721 [95% CI: 1.418, 2.090]), with a significant difference (*p* < 0.05). The data suggest that the risk of developing RA increases with increasing levels of IBI.

**Table 2:** Odds ratio of RA according to IBI in different models.

	RA OR (95% CI), *p*		
	Model 1	Model 2	Model 3
IBI	1.003 (1.003, 1.004) <0.001	1.002 (1.001, 1.003) <0.001	1.002 (1.001, 1.003) <0.001
IBI quartile			
Q1	1	1	1
Q2	1.554 (1.295, 1.864) <0.001	1.107 (0.901, 1.361) 0.334	1.093 (0.888, 1.346) 0.400
Q3	2.266 (1.909, 2.691) <0.001	1.321 (1.083, 1.610) 0.006	1.319 (1.079, 1.612) 0.007
Q4	3.471 (2.948, 4.087) <0.001	1.723 (1.422, 2.087) <0.001	1.721 (1.418, 2.090) <0.001

Model 1 was adjusted for none.Model 2 was adjusted for age, sex, race, education, marital status, family poverty-to-income ratio, body mass index, smoking status, drinking alcohol status, stroke, cerebrovascular disease, diabetes, hypertension.Model 3 was adjusted for age, sex, race, education, marital status, family poverty-to-income ratio, body mass index, smoking status, drinking alcohol status, stroke, cerebrovascular disease, diabetes, hypertension, glycated hemoglobin, alanine aminotransferase, aspartate aminotransferase, blood urea nitrogen, total cholesterol, high-density lipoprotein.

### 3.3 The nonlinear relationship between RA and IBI

**Figure 2:**
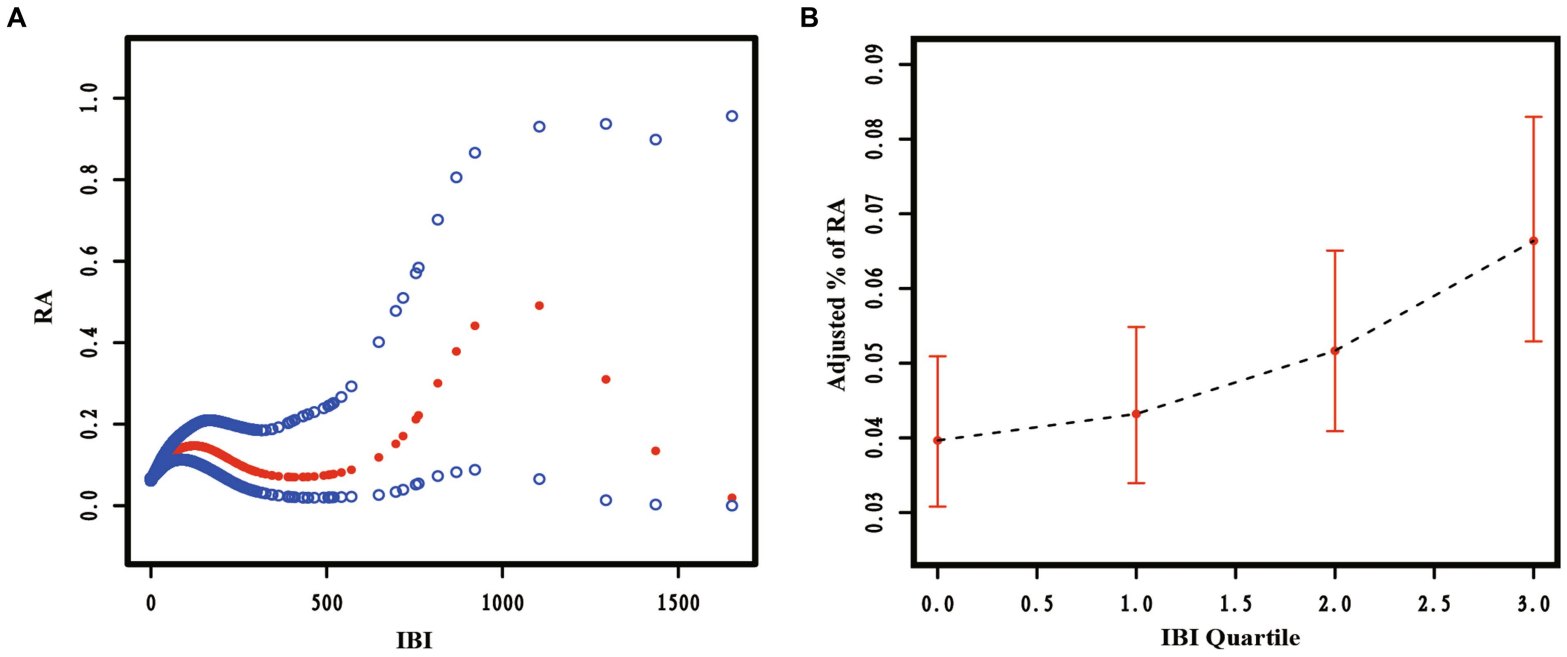
The restricted cubic spline curve of the association between Inflammatory Burden Index (IBI) and Rheumatoid Arthritis (RA). Relationship between IBI as a continuous variable **(A)** and IBI quartiles **(B)** with RA.

This study analyzed the potential non-linear relationship between the risk of RA prevalence and IBI using RCS curves. The results, shown in Figure 2, indicate a significant non-linear relationship between IBI level and RA prevalence (*p* < 0.05). The prevalence of RA increased as the level of IBI increased, suggesting a positive correlation between RA and IBI levels.

### 3.4 Subgroup analyses and interactions to test the association between IBI and the risk of RA

**Figure 3:**
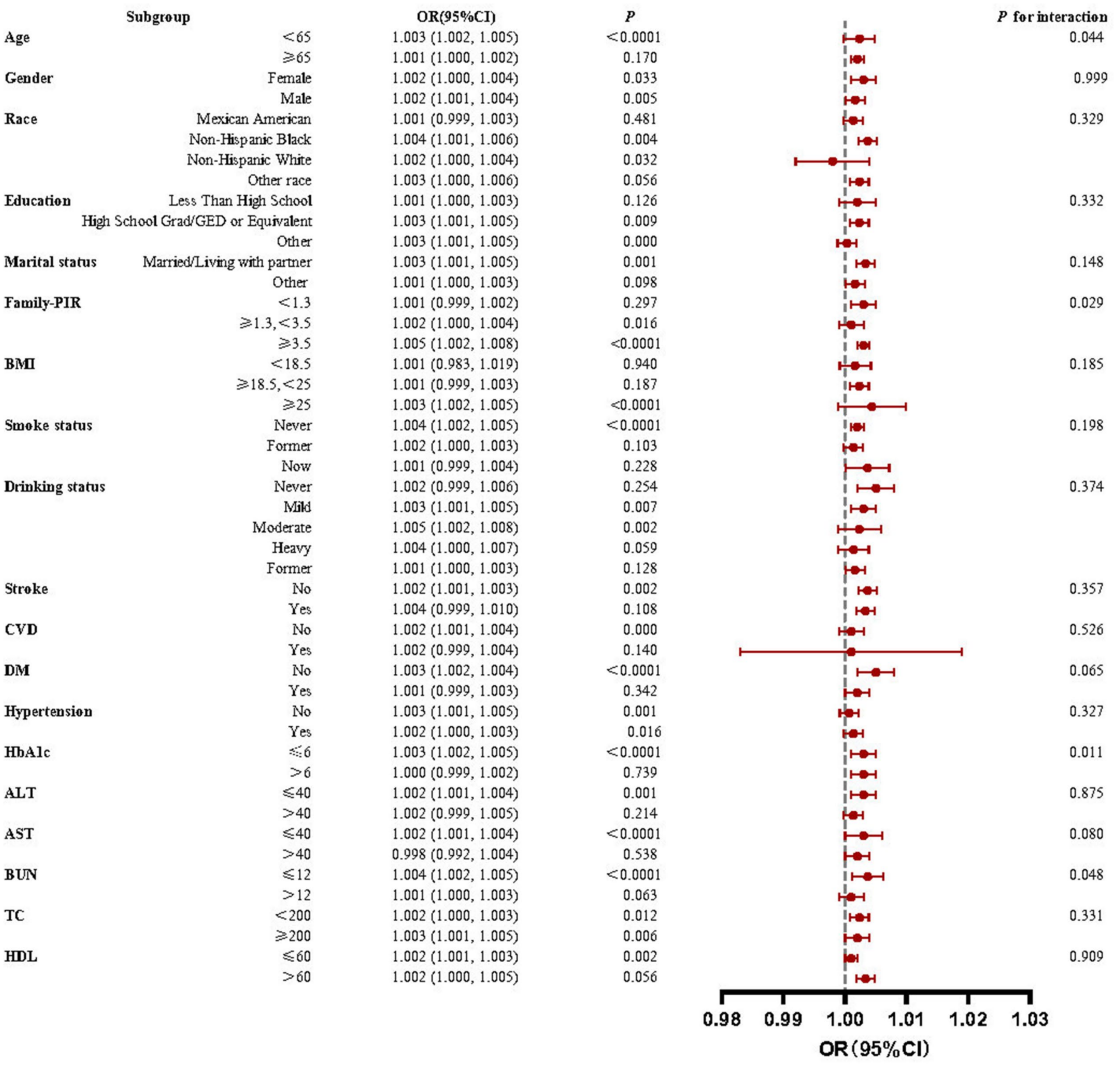
Forest plots for subgroup analyses and interaction tests of the relationship between IBI and RA risk. A significant positive correlation between IBI levels and RA prevalence was observed in most subgroups with stable results.

This study used subgroup analyses to generate effect estimates for each group. Figure 3 demonstrates that the positive correlation between RA prevalence and IBI scores was statistically significant in most subgroups. However, in a portion of subgroups, the positive association was insignificant. This positive association was not statistically significant (*p* > 0.05) among participants aged ≥65, partial race, low education level, household poverty-to-income ratio < 1.3, BMI < 25, partial disease (stroke, CVD, diabetes), HbA1c > 6, ALT > 40, AST > 40, BUN > 12, and HDL > 60.

Interaction tests revealed that the majority of confounders in the association between IBI scores and RA did not significantly interact with this association (*p* > 0.05 for all interactions). Among the multiple covariates, only household poverty-to-income ratio, HbA1c, and BUN influenced this relationship.

### 3.5 Association between IBI and all-cause mortality in RA

The study analyzed the relationship between IBI and RA all-cause mortality using several models adjusted for different covariates. The results of the regression analyses are presented in Table 3, indicating a positive and significant correlation between all-cause mortality and IBI score in all three models (*p* < 0.05). When converting IBI scores to quartiles, the highest quartile was associated with increased all-cause mortality in RA compared to the lowest quartile used as the reference. Model 1, which did not adjust for any covariates, showed a significantly higher hazard ratio (HR) for all-cause mortality in participants in the highest IBI quartile compared to those in the lowest quartile (HR for Q2, 1.272 [95% CI: 0.932, 1.735]; for Q3, 1.435 [95% CI: 1.072, 1.922]; for Q4, 1.650 [95% CI: 1.250, 2.178]). In Model 2, all-cause mortality was 42.4% higher in the highest quartile than in the lowest quartile (Q4, 1.424 [95% CI: 1.043, 1.845]), which was a statistically significant difference. In model 3, after adjusting for all covariates, it was still observed that all-cause mortality was 46.4% higher in the highest quartile group (Q4, 1.464; 95% CI: 1.068, 2.007) than in the lowest quartile group. The correlation between the two was significant in all models (*p* < 0.05), suggesting that increased IBI scores are strongly associated with increased all-cause mortality in RA.

**Table 3:** Hazard ratio of all-cause mortality according to IBI in different models.

	All-cause mortality HR (95% CI), *p*		
	Model 1	Model 2	Model 3
IBI	1.001 (1.001, 1.002) 0.002	1.003 (1.002, 1.004) <0.001	1.003 (1.002, 1.005) <0.001
IBI quartile			
Q1	1	1	1
Q2	1.272 (0.932, 1.735) 0.130	0.943 (0.669, 1.330) 0.740	0.968 (0.683, 1.370) 0.853
Q3	1.435 (1.072, 1.922) 0.015	0.978 (0.704, 1.359) 0.900	1.034 (0.740, 1.443) 0.846
Q4	1.650 (1.250, 2.178) <0.001	1.424 (1.043, 1.945) 0.026	1.464 (1.068, 2.007) 0.018

Model 1 was adjusted for none.Model 2 was adjusted for age, sex, race, education, marital status, family poverty-to-income ratio, body mass index, smoking status, drinking alcohol status, stroke, cerebrovascular disease, diabetes, hypertension.Model 3 was adjusted for age, sex, race, education, marital status, family poverty-to-income ratio, body mass index, smoking status, drinking alcohol status, stroke, cerebrovascular disease, diabetes, hypertension, glycated hemoglobin, alanine aminotransferase, aspartate aminotransferase, blood urea nitrogen, total cholesterol, high-density lipoprotein.

### 3.6 Non-linear relationship between IBI and all-cause mortality in RA

**Figure 4:**
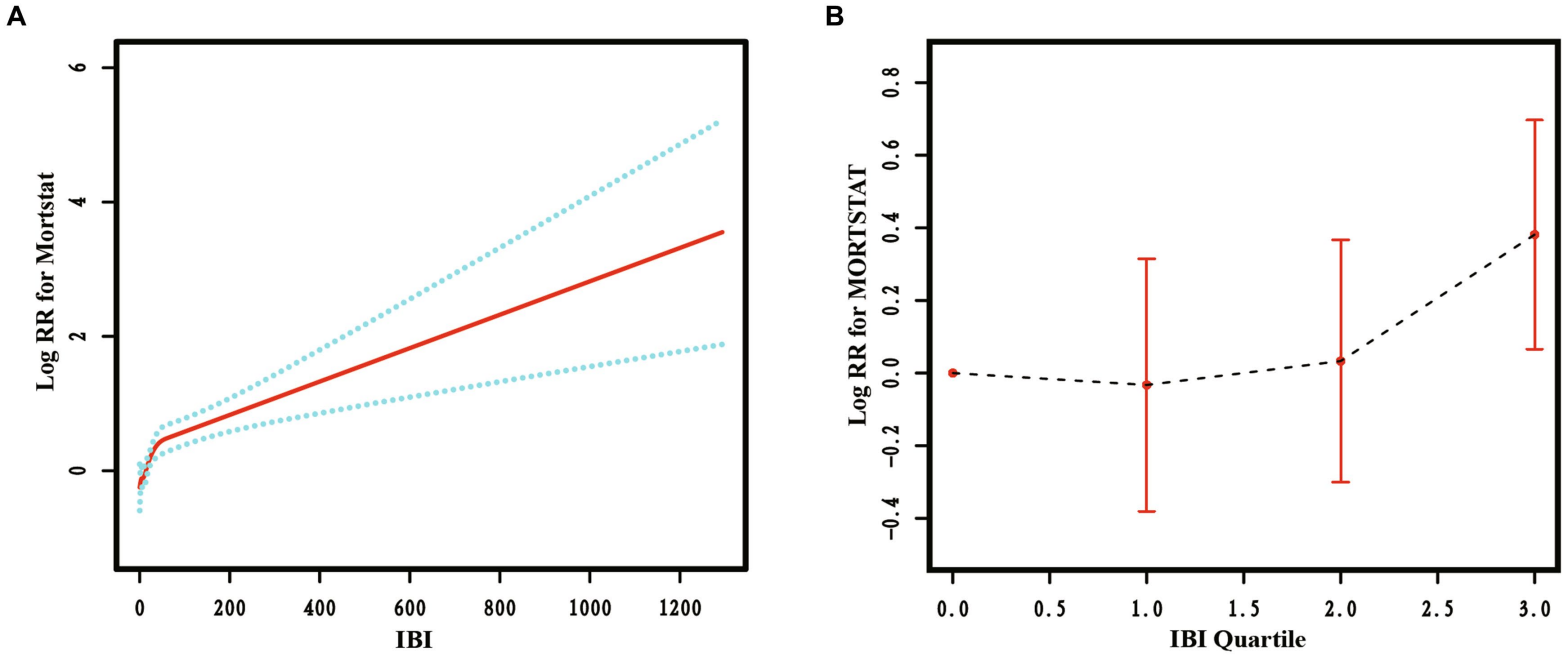
The restricted cubic spline curve of the association between IBI and all-cause mortality in RA. Relationship between IBI as a continuous variable **(A)** and IBI quartiles **(B)** with all-cause mortality in RA.

RCS curves were used to analyze the non-linear relationship between IBI and RA all-cause mortality. The results are presented in Figure 4, indicating a significant non-linear relationship between IBI levels and RA all-cause mortality (*p* < 0.05). As the IBI level increased, the RA all-cause mortality rate also increased, exhibiting an inverted J-shape.

### 3.7 Subgroup analyses and interaction tests for the association between IBI and all-cause mortality in RA

**Figure 5:**
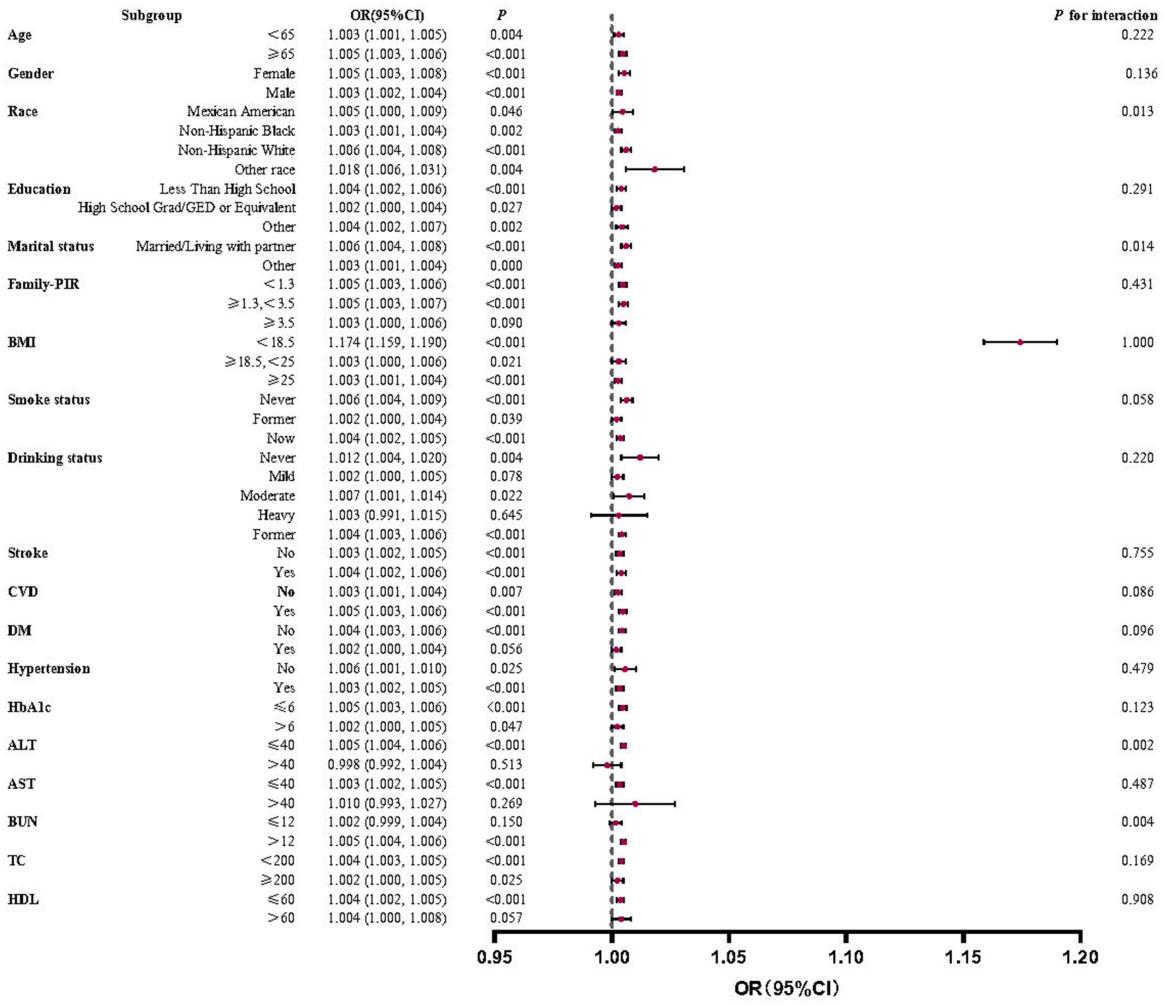
Forest plots for subgroup analyses and interaction tests of the relationship between IBI and all-cause mortality in RA. A statistically significant positive correlation between IBI score and all-cause mortality in the vast majority of subgroups with stable results.

This study conducted subgroup analyses to estimate the impact of different subgroups on the association between IBI and all-cause mortality in RA. The results in Figure 5 show a statistically significant positive correlation between IBI score and all-cause mortality in the vast majority of subgroups. However, in a minor subgroup, the positive correlation was not significant. The positive association was not statistically significant among those with a household PIR ≥3.5, mild or heavy alcohol consumption, diabetes mellitus, ALT > 40, AST > 40, BUN ≤ 12, and HDL > 60 (*p* > 0.05).

Interaction tests showed that the overwhelming majority of covariates did not significantly interact with each other for the positive correlation between IBI scores and RA all-cause mortality (*p* > 0.05). Among multiple subgroups, only race, marital status, ALT, and BUN impacted this correlation.

## 4 Discussion

This research first investigates the association between IBI scores and the risk of RA and all-cause mortality in an American adult population. Statistically significant positive associations were observed between IBI and both the risk of RA and its all-cause mortality. The higher the IBI, the higher the risk of RA and its all-cause mortality. The relationship between IBI and both outcomes was analyzed separately using RCS curves, and it was found that the positive associations were consistently non-linear across all models. Subgroup analyses and interaction tests confirmed the stability of all correlations.

As a newly proposed, more comprehensive inflammatory indicator, IBI presents superior prospects for application and clinical significance compared to traditional inflammatory markers. Proponents of IBI metrics believe it is a more reliable biomarker that can predict poor prognosis in cancer patients and can be applied to assess the inflammatory burden of different cancers (6). IBI can provide predictive stratification of patients with localized advanced gastric cancer as a pathological adjunct to their prognostic assessment (11). A high preoperative IBI score may be an independent risk factor for surgical site infection in oesophageal cancer and a useful predictor of prognosis and surgical site infection in oesophageal cancer patients after oesophagectomy (12). Similarly, IBI can also predict poor prognosis in patients with hepatocellular carcinoma after hepatectomy, offering a higher and more accurate prognostic value compared to existing markers (13). High IBI was consistently associated with increased complications and mortality after surgery in advanced gastric cancer treated with multimodal therapy, emerging as the only significant predictive variable of postoperative outcomes (14). Therefore, IBI can provide a patient-specific and personalized reference for outcome monitoring, prognostic assessment of different cancers, and obtaining more precise medical treatment.

For other diseases, studies have also begun to focus on the new indicator of IBI, which is a more reliable marker for predicting poor outcomes in acute ischaemic stroke patients treated with endovascular thrombectomy, and high levels of IBI are associated with an increased risk of poor outcomes (15). High levels of IBI have also been linked to poorer prognoses in patients with aneurysmal subarachnoid hemorrhage and are independently associated with an increased incidence of pneumonia and deep vein thrombosis (7). The increased risk of all-cause mortality in adults over 45 years of age is associated with high levels of IBI, suggesting that controlling inflammation is a critical factor in reducing the risk of premature death in this demographic (16).

This study illustrates that IBI serves as a comprehensive indicator of RA patients’ immune and inflammatory status by integrating data on neutrophils, CRP, and lymphocytes. Although RA is a chronic autoimmune disease, it is frequently caused by chronic inflammation throughout the body that leads to a variety of severe systemic symptoms and thus increased mortality (1). The development of arthritic symptoms in RA patients is due to a complex interplay of cells, including lymphocytes and neutrophils. Lymphocytes, primarily infiltrating cells in the affected joints, elicit specific immune responses in associated tissues such as lymph nodes (17). Abnormal humoral immunity in RA patients promoted the over-activation of auto-reactive T and B cells, leading to significant antibody production and the formation of immune complexes in the synovial tissue, exacerbating the disease progression (18). T-lymphocytes transform protective tissue cells into agents of tissue destruction, significantly influencing the disease’s severity and patients’ quality of life (5). Multiple T cells and corresponding pathways mediate inflammatory processes, further promoting RA (19). Activated B lymphocytes are critical for the progression of RA due to their role in autoantibody production. In peripheral blood samples from RA patients, lymphocyte counts may vary with disease activity and response to treatment (18).

Neutrophils are pivotal in RA pathogenesis, among the first immune cells to respond at the inflammation site. They activate antigen-presenting cells and participate in the production of pro-oxidant mediators and lytic enzymes (20). Their role in producing reactive oxygen species contributes to endothelial dysfunction and subsequent tissue damage, which are hallmarks of ongoing inflammation (21). In RA patients, hypoxia and increased glycolysis enhance neutrophil activation, which produces neutrophil extracellular traps, which are implicated in the autoimmunity seen in RA. Activated neutrophils also engage in cartilage destruction and bone erosion and secrete a variety of immune mediators such as IL-1, IL-6, and others that lead to acute and persistent inflammation (1, 18, 22). The interaction between these cell types contributes to the disease’s complexity. Neutrophils can influence T cell responses through their secreted products and physical interactions, while activated T cells can recruit and activate more neutrophils, creating a vicious cycle of inflammation. Both genetic predispositions and environmental factors, such as smoking, can influence the roles and efficacy of these immune cells in RA (5). The heterogeneity in patients’ genetic backgrounds and environmental exposures contributes to the variability in disease presentation and progression, affecting how neutrophils and lymphocytes function in the disease context.

CRP, as an acute-phase protein that monitors the progression of the disease and the efficacy of therapy in RA, is an essential protein in the assessment of inflammation (23). Patients have elevated serum levels of CRP due to the systemic inflammatory state, which also promotes further inflammation. In RA, higher neutrophil counts suggest the presence of a persistent non-specific inflammatory process, while lower lymphocyte counts indicate a relatively compromised immune system. An elevated ratio of the two indexes indicates an enhanced pro-inflammatory innate response and an underlying defect in anti-inflammatory lymphocyte activity. The IBI multiplies this by the CRP, with larger values indicating a higher level of inflammation in the patient.

Given the complexity of RA’s pathogenesis and its systemic impacts, various extra-articular manifestations and comorbidities significantly contributed to increased patient mortality. For example, the involvement of cardiovascular and respiratory systems due to systemic inflammation significantly increases the risk of morbidity and mortality in RA patients (3, 24). The underlying inflammatory mechanisms of RA share common pathways with cardiovascular diseases, including shared inflammatory mediators and endothelial dysfunction, which exacerbate the patient’s overall health burden (24). Approximately 35% of RA patients experience respiratory complications, representing a major cause of morbidity and mortality in this population (25). The use of immunosuppressive medications in RA management increases susceptibility to infections, particularly respiratory infections, which are significant contributors to increased mortality in these patients (3). Thus, RA’s systemic nature and extra-articular manifestations necessitate a comprehensive management approach to mitigate these risks. Studies have shown that all-cause mortality is 50% higher in RA patients than in the general population, underscoring the severe impact of this disease on patient outcomes (26). Therefore, leveraging IBI as a new comprehensive inflammation indicator provides crucial insights into the inflammatory and immune status of RA patients, offering potential pathways for intervention and better management of the disease (16).

To the best of our knowledge, the present study is the first to investigate the relationship between IBI indicators and the risk of developing RA and to further investigate the relationship between IBI and all-cause mortality in RA. The main advantages of this study are the relatively large sample size and national representativeness using data from the NHANES database. In addition, the use of multiple data analysis methods to examine the relationship between IBI indicators and RA makes the results of this study more credible. However, there are unavoidable limitations to this study. First and foremost, this study is a population-based cross-sectional study, which can only provide the correlation between IBI and RA, and does not infer a causal relationship. Second, we could only partially exclude the influence of other possible unmeasured and confounding factors during the analysis, and no treatment programs were included as covariates. Third, there needed to be more opportunity to examine the dynamics of IBI throughout the disease, as the data came from a database. Fourth, some of the confounders in the study may have been subject to participant recall bias, which could have affected the study results. Fifth, the NHANES database has limitations in diagnosing RA as its results are derived from self-reported data rather than a physician’s clinical diagnosis. Finally, the data in this study came from the US population, and it is unclear whether the same results can be extrapolated to other ethnic groups. Whether our conclusions are ultimately applicable to the entire human population would require a larger survey or randomized controlled trial to confirm. Or the entire literature needs to be retrieved for Meta-analysis or bibliometric analysis. Taken together, the causal relationship needs to be confirmed by more prospective studies and relevant experimental studies.

## 5 Conclusion

The results of this study indicate that there is a positive correlation between IBI and both RA prevalence and all-cause mortality and that this positive correlation is non-linear and stable. This suggests that we should be aware of the level of inflammation represented by the IBI metric, and that the lower the level of inflammation in the body, the lower the risk of both RA prevalence and all-cause mortality. Therefore, controlling inflammation is crucial. Nevertheless, our findings warrant further prospective studies to explore the mechanisms involved.

## Data Availability

The original contributions presented in the study are included in the article/supplementary materials, further inquiries can be directed to the corresponding authors.

## Glossary

NHANES: National Health and Nutrition Examination Survey
RA: Rheumatoid arthritis
IBI: Inflammatory Burden Index
CRP: C-reactive protein
PIR: Poverty-to-income ratio
CVD: Cerebrovascular disease
DM: Diabetes mellitus
HbA1c: Glycated hemoglobin
ALT: Alanine aminotransferase
AST: Aspartate aminotransferase
TC: Total cholesterol
HDL: High-density lipoprotein
LDL: Low-density lipoprotein
BUN: Blood urea nitrogen
BMI: Body mass index
RCS: Restricted cubic spline
OR: Odds ratio
CI: Confidence Interval
HR: Hazard ratio
